# Does Change Predict Change in the CHANGE Intervention Study?

**DOI:** 10.1093/geroni/igaa057.1204

**Published:** 2020-12-16

**Authors:** Scott Maitland, Paula Brauer, David Mutch, Dawna Royall, Doug Klein, Angelo Tremblay, Caroline Rhéaume, Khursheed Jeejeebhoy

**Affiliations:** 1 University of Guelph, Guelph, Ontario, Canada; 2 University of Alberta, Edmonton, Alberta, Canada; 3 Laval University, Québec, Quebec, Canada; 4 University of Toronto, Toronto, Ontario, Canada

## Abstract

Does change in one lifestyle factor (e.g., exercise/fitness) help explain change in another factor or process (i.e., dietary behavior or cardiovascular risk)? The current modeling was a secondary analysis of a primary care feasibility study of individualized lifestyle (diet and exercise) treatment of metabolic syndrome (n=293; mean age = 59yrs) that achieved 19% reversal over one year. Diet quality was assessed by the Healthy Eating Index (HEI) (2005 Canada); while fitness was assessed by several measures (VO2max, flexibility, curl-ups, push-ups). Three occasions (i.e., baseline, 3-, and 12-month) were examined using latent change score and latent growth curve models (in AMOS) to assess whether changes in one domain predicted changes in the remaining domains: (1) diet (measured by HEI or latent construct); (2) fitness (measured by VO2max percentiles or latent construct); and, (3) 10-year risk of cardiovascular disease (by Procam Risk score). Results showed significant improvement in all three domains separately during the intervention, with greater change between baseline and 3-month assessment and continued change between 3- and 12-months. Initial status variables on observed constructs were moderately positively correlated and change in dietary behavior was significantly related to change in fitness levels, but neither were significantly related to change in the 10-year risk of cardiovascular disease. In addition, the associations between change in diet and changes in fitness were inconsistent baseline to 3 months, and 3-12 months. These results offered new insight on relationships among interventions in a behavioural counselling program which can inform future programming.

